# Investigation and Improvement of Bond Performance of Synthetic Macro-Fibres in Concrete

**DOI:** 10.3390/ma13245688

**Published:** 2020-12-13

**Authors:** Mantas Garnevičius, Linas Plioplys, Pui-Lam Ng, Shaohua Chu, Viktor Gribniak

**Affiliations:** 1 Department of Steel and Composite Structures, Vilnius Gediminas Technical University (Vilnius Tech), Saulėtekio av. 11, LT-10223 Vilnius, Lithuania; Mantas.Garnevicius@vgtu.lt (M.G.); Linas.Plioplys@vgtu.lt (L.P.); 2 Laboratory of Innovative Building Structures, Vilnius Tech, Saulėtekio av. 11, LT-10223 Vilnius, Lithuania; irdngpl@gmail.com; 3 Laboratory of Concrete Technologies, Vilnius Tech, Saulėtekio av. 11, LT-10223 Vilnius, Lithuania; 4 Department of Civil Engineering, The University of Hong Kong, Pokfulam, Hong Kong 999077, China; shchu@connect.hku.hk

**Keywords:** synthetic macro-fibres, bond, pull-out test, colloidal silica, steel micro-fibres

## Abstract

Strength and stiffness are the key parameters characterising the bond performance of fibres in concrete. However, a straightforward procedure for estimating the bond parameters of a synthetic macro-fibre does not exist. This study employs pull-out tests to investigate the bond behaviour of synthetic macro-fibres. Two types of macro-fibres available in the market were investigated. A gripping system was developed to protect the fibres from local damage. The experimental campaign consisted of two stages. At the first stage, 32 concrete specimens were manufactured for performing 96 pull-out tests (three fibre samples were embedded in each cube perpendicular to the top surface and two sides). Two types of macro-fibres with either 10 or 20 mm embedment length were tested. The obtained load–displacement diagrams from pull-out tests demonstrate that the bond performance (characterised by the strength and deformation modulus) of the “top” fibres is almost 20% weaker than fibres positioned to the side surfaces. At the second stage, one type of macro-fibre was chosen for further experimentation of the feasibility of improving the bond performance through the use of colloidal silica or steel micro-fibres. This investigation stage employed an additional 36 concrete specimens. The use of steel micro-fibres was found to be an efficient alternative. The success of this solution requires a suitable proportioning of the concrete.

## 1. Introduction

In contrast to bar reinforcement, fibres offer good potential to serve as dispersed reinforcement in concrete construction in the commonplace. The concept of the structural application of fibrous reinforcement had been laid down since the last century [1]. Compared to conventional reinforced concrete, fibre-reinforced concrete (FRC) can be superior in terms of crack resistance, ductility, and residual stiffness of the cracked elements [2,3]. The fibres have a significant role in controlling the widening and propagation of cracks. Structurally, the fibres prevent crack proliferation by transferring tensile stresses across the crack to tension along the fibres, as well as to the bond with surrounding concrete [4,5]. This crack bridging mechanism can increase the energy absorption in the post-crack regime and improve the ductility of FRC [6]. In structural engineering design, the RILEM recommendation [7] considers FRC as a homogeneous substance with modified material properties differing from conventional concrete. This simplified approach enables neither estimating the fibre interaction mechanisms nor identifying the causes of alteration of mechanical properties of FRC. Accurate analysis of FRC as a composite material is complicated by the variety of sources and physical parameters of fibres and concrete mix proportions [8]. The type, length, surface texture, and geometrical features of fibres, mechanical properties of the concrete, content of fibres in concrete, and maximum nominal size of aggregates influence the bond performance of the fibre as dispersed reinforcement [9,10]. Recent publications [11,12,13,14,15,16,17,18,19] demonstrated that combinations of synthetic and steel fibres are efficient in structural applications. However, there is generally the concern of inefficiency in load resistance of synthetic macro-fibres in structural use [19,20,21]. With the recent advances of synthetic materials, the tensile strength capacity of synthetic fibres can be comparable to that of metallic reinforcing bars. This development has broadened the application scope of synthetic fibres, especially in combined use with steel fibres [22,23].

It is known that the structural properties of FRC are highly dependent on the bond characteristics of fibres. Juhász [6] pointed out the essential difference between the interaction mechanisms of steel and synthetic fibres with concrete: while steel fibres pull-out from the matrix, synthetic fibres bridge the crack. Nevertheless, there is no universal methodology for evaluating the bond parameters of a synthetic macro-fibre [24,25]. To address this gap, the bond behaviour of synthetic macro-fibres in concrete is the targeted object of this research. The bond strength and deformation modulus together define the bond behaviour and indicate the interaction mechanism that is responsible for the residual strength of FRC—the primary property that describes the efficiency of structural application of the fibres. This study employs pull-out tests [26] to investigate the bond behaviour of synthetic macro-fibres, with the use of a gripping system developed by the authors’ research team to prevent stress localisation and protect the fibres from local damage. Two types of synthetic fibres available in the market were included in the experimental campaign. The maximum pull-out force and corresponding slip, the total deformation energy, the maximum slip displacement, and the failure behaviour were studied and compared. One of the fibre types was rationally chosen for further analysis of the feasibility to improve the bond performance. To this end, adequate consideration was given to the strength of the fibre material necessary to activate the bond-slip mechanism. Innovative attempts to improve the bond performance were explored on this basis. These included the use of colloidal silica and the variation in the volumetric content of steel micro-fibres as the experimental parameters.

In the conventional practice, the employment of colloidal silica and steel micro-fibres are not often adopted as solutions for improving the bond performance of synthetic macro-fibres. Densification of the concrete at the fibre–concrete interface [27,28] has provided a rationale for exploring the former solution. The effect of micro-fibres confining microstructural deformation of the concrete [29,30] in the proximity motivated the latter solution. The steel micro-fibres develop a similar mechanism to macro-fibres as dispersed reinforcement to benefit the structural properties of FRC [31,32] but on a micro-scale. However, the presence of rigid steel fibres substantially reduced the workability of fresh concrete [33,34]. That has been a crucial reason limiting the use of steel fibrous concretes [35,36]. In this study, appropriate measures of concrete mix adjustment and optimisation were adopted to cope with the different volumetric contents of steel micro-fibres. The research results provide a valuable reference for developing efficient FRC.

## 2. Experimental Campaign

The testing program consists of two stages. In the first stage, pull-out tests were performed to determine the bond performance of two types of synthetic macro-fibres available in the market. In the second stage, one type of fibre was chosen for further experimentation of the feasibility of improving the bond performance. Remarkably, two non-conventional solutions were explored, namely, the use of colloidal silica and the use of steel micro-fibres.

**Figure 1:**
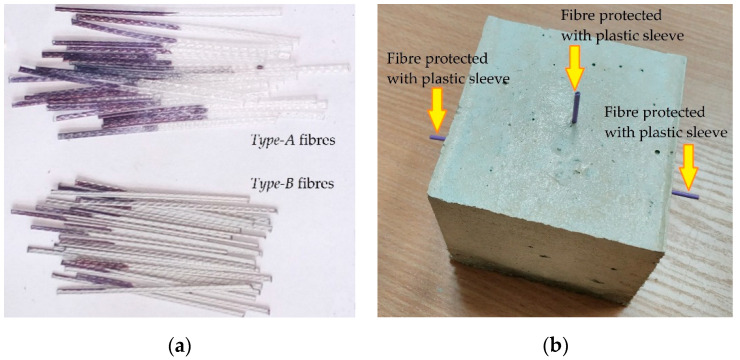
Specimens for pull-out test: (**a**) *Type-A* and *Type-B* fibres highlighting the embedment length; (**b**) a typical cube with embedded fibres.

**Figure 2:**
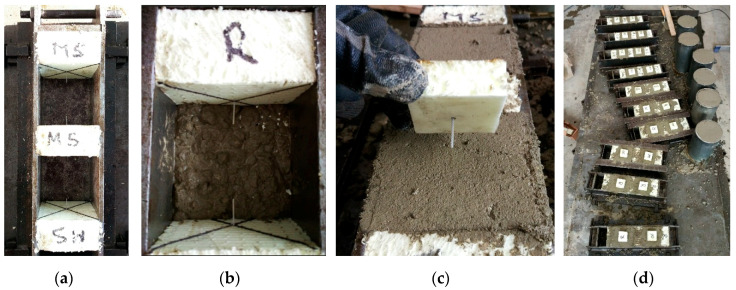
Preparing pull-out test specimens: (**a**) steel moulds with expanded polystyrene; (**b**) mould partially filled with concrete; (**c**) a manual placement of the top fibre; (**d**) specimen series after concreting.

Two fibre types (designated as *Type-A* and *Type-B*) were included in the experimentation. Figure 1a shows these fibres. *Type-A* fibre had a length of 45 mm and equivalent diameter of 0.9 mm; it had tensile strength of 465 MPa and elastic modulus of 3350 MPa. *Type-B* fibre had a length of 40 mm and equivalent diameter of 0.7 mm; it had tensile strength of 500 MPa and elastic modulus of 6000 MPa. Comparatively, *Type-A* fibre had a deeper texture of surface embossment than *Type-B* fibre. The fibres were partially embedded in 100 mm cubes for pull-out testing; two bonding lengths (10 and 20 mm) were considered. The test campaign encompassed four sets of specimens from the combination of two fibre types and two embedment lengths. Each set was experimentally investigated by testing with eight cubes. In total, 32 cube specimens were prepared. Three fibres (one on the top surface and two on the opposite side surfaces) were inserted in each cube, as shown in Figure 1b. The fibres were marked to indicate the correct embedment depth (Figure 1a). Expanded polystyrene was placed in the moulds (Figure 2) with fibres inserted before concreting (Figure 2a) to ensure the exact position of the “side” fibres (Figure 2b), whereas the “top” fibre was inserted manually into the specimen when the concrete was fresh (Figure 2c). The concrete was compacted by a vibration plate (Figure 2d) to achieve proper compaction. After curing and demoulding, the length of the unbonded part of the fibre outside the specimen concrete was measured to verify the actual embedment length.

All cube samples were produced from one concrete batch. Proportions of concrete are expressed in terms of contents of ingredients per cubic metre in the following: 300 kg of type CEM I 42.5 R cement, 165 kg of water, 100 kg of limestone powder, 787 kg of 0/4 mm sand, and 988 kg of 4/16 mm crushed aggregates; 0.75% (by mass of the cement) of superplasticiser *Mapei Dynamon XTend*. The average slump of the fresh concrete was measured to be 180 ± 35 mm. Average compressive strength of the 100 mm concrete cubes measured at the 28-day age and the testing date of 153-day age was equal to 39.14 and 46.16 MPa, respectively.

**Figure 3:**
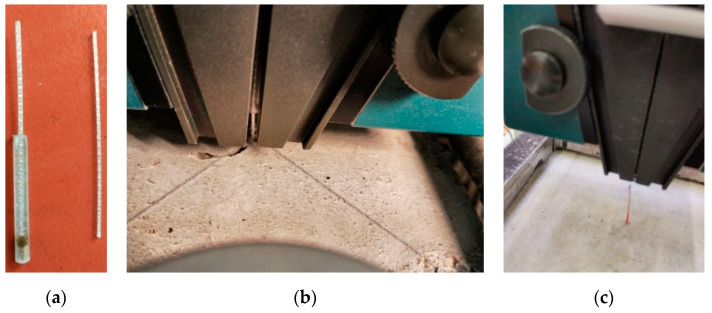
Preliminary pull-out tests of synthetic macro-fibres [37]: (**a**) fibres protected with plastic sleeve; (**b**) failure of the fibre samples due to slip of the protection sleeve; (**c**) loss of the cohesive contact with the grips.

**Figure 4:**
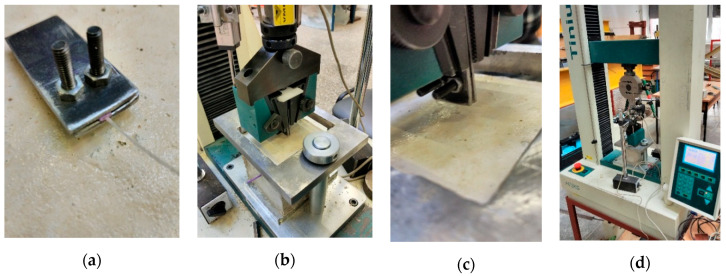
Gripping system: (**a**) steel clamps; (**b**) inventory grips by testing machine; (**c**) the steel clamps in the grips; (**d**) electromechanical testing apparatus *H75KS*.

The former attempts to investigate the bond performance of synthetic macro-fibres [37] demonstrated the necessity of a reliable protection system for the polymeric fibre samples. Either loss of the cohesive contact with grips or slip of the protection sleeves would defeat the pull-out test and thus should be avoided. Figure 3 shows the typical examples of the preliminary tests. These unsuccessful results motivated the development of a gripping system to protect the fibres from local damage. In the same manner as applied previously [37], plastic sleeves (Figure 3a) were used for preserving the unbonded part of the fibres from damage. The mechanical properties (flexibility and friction coefficient) of the sleeves were chosen using the trial-and-error procedure. Adjustable steel clamps (Figure 4a) were additionally introduced to fix the protected fibre, preventing the localisation of stresses induced by the gripping system of the tension apparatus (Figure 4b–d).

**Figure 5:**
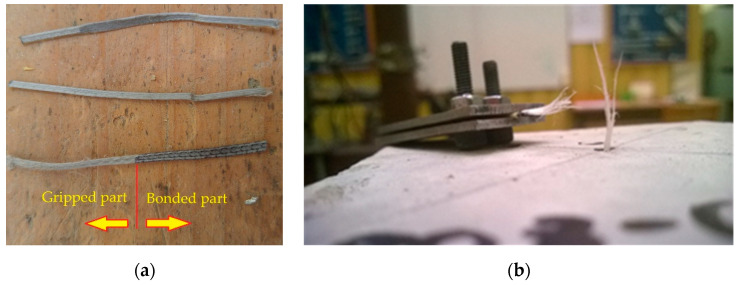
Pull-out test results: (**a**) tested fibre samples; (**b**) broken fibre protected with a plastic sleeve.

The testing apparatus was a 75 kN capacity electromechanical machine *H75KS* (*Tinius Olsen*, Norway). The fibre under testing was loaded in a deformation-control manner with 0.8 mm/min loading rate. A 2 kN load cell was employed to measure the applied load. Higher precision of measurement could be attained by using such a relatively small capacity load cell. Vertical displacements of the grips were monitored using linear variable differential transformers (LVDT), as shown in Figure 4d; an additional LVDT was installed to monitor the vertical movements of the cube specimen. Readings from all devices (LVDT and the load cell) were acquired every second through the signal processing equipment Almemo 2890-9 (manufacturer: *Ahlborn Mess- und Regelungstechnik GmbH*, Germany) and recorded by a workstation computer. Figure 5a depicts examples of the tested samples of *Type-B* fibres.

## 3. Results of First Stage of Experimentation

The pull-out specimens manifested three types of failure: (I) pull-out of fibre from the concrete (Figure 5a), which was taken as the normal failure mode; (II) failure of a fibre (Figure 5b), which signified premature breakage and should be excluded from the valid test results; (III) damage before the test (incoherent result), which should be excluded from the valid test results. Among these failure types, the latter mode is characteristic of the bond behaviour of the “top” fibres. That can be explained by the weakening of the bond due to sedimentation of the fresh concrete [38]. In all cases, the bond properties of the fibres inserted at top surfaces were inferior to those of the fibres positioned at the side surfaces. Moreover, the forces required to pull out the “top” fibres from the concrete were more scattered compared to the “side” samples. Thus, only the test results from the “side” fibres are employed for further analysis.

Table 1 summarises the test results including the embedment length *l_e_*, the maximum pull-out force **P***_max_*, the corresponding slip displacement *u_P_*, the total deformation energy *δ* (evaluated as the area beneath the load–displacement curve up to the maximum slip displacement), and the corresponding slip displacement *u_δ_*. This table only displays results of the fibres satisfactorily embedded in the concrete, i.e., the orientation of the fibre was normal to the concrete surface with negligible discrepancies in the length *l_e_* from the target value and without evident bond defects.

From Figure 5a, the pulled-out fibre surface was adhered with traces of cement paste, which accords with the expectation. Table 1 demonstrates that the energy dissipation capacity (the displacement energy *δ*) of the *Type-A* fibre was almost twice of that of the *Type-B* fibre. Among these two types of fibres, the corresponding average energy *δ* released for the length *l_e_* = 10 mm was respectively equal to 1077.4 and 533.9 Nmm; whereas for the length *l_e_* = 20 mm, the corresponding energy value was equal to 2671.7 and 1484.1 Nmm, respectively. Only the results of completely pulled out fibres were included in the averaging (the results of incompletely extracted fibres as highlighted in grey colour in Table 1 were excluded from the averaging).

**Figure 6:**
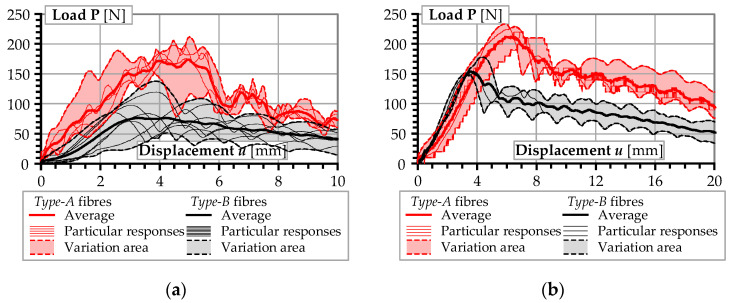
Load–displacement diagrams of the pull-out tests: (**a**) fibres with embedment length *l_e_* = 10 mm; (**b**) *l_e_* = 20 mm.

Figure 6 shows the load–displacement diagrams of completely pulled out fibres. The scattering of fibre pull-out test results is in line with the behavioural norm [39,40,41], even though the specimens were prepared in good workmanship and quality. The scattered phenomenon is particularly true for the low stiffness synthetic macro-fibres in contrast to the more stiff metallic fibres and anchors [6,42,43]. Bond defects due to the heterogeneity of concrete were another possible cause of the observed scatter. For these reasons, testing of multiple specimens was required.

Figure 6 reveals considerable differences in resistance of the alternative fibres. The increase in the embedment length *l_e_* (from 10 to 20 mm) had distinct effects on the bond behaviour of different fibre types. Comparing the maximum pull-out forces (Table 1), the value of **P***_max_* for *Type-A* fibre was also on average almost twice of that for *Type-B* fibre. This was due to the larger cross-sectional area of *Type-A* fibre as well as the deeper texture of surface embossment that led to better interlocking with the surrounding concrete [44]. In view of its more superior mechanical properties, *Type-A* fibre was chosen for examining the feasibility of improving the bond performance.

## 4. Results of Second Stage of Experimentation

In the second stage of the study, an additional 36 concrete specimens were employed. There were two solutions attempted for improving the bond performance of the chosen synthetic macro-fibres. Firstly, the effectiveness of colloidal silica as a chemical additive was investigated. Secondly, the volumetric content of steel micro-fibres was investigated. The procedures of pull-out tests were the same as the first stage and are described in Section 2. For the reason explained earlier, only the results of the “side” fibres were considered.

### 4.1. Use of Colloidal Silica

During the first attempt, a colloidal silica with the trademark *TCAM10* supplied by TechConcrete was investigated as a chemical additive for potentially improving the bond properties. This additive was a liquid containing up to 50% of silicon dioxide, and it densifies the microstructure of concrete by reacting with the portlandite to form additional calcium-silicate-hydrate in the interstitial pores of hardened concrete [27,28,45]. The use of *TCAM10* is expected to improve the bond of fibres with the cementitious matrix. Table 2 describes the mix proportions of the concretes with and without colloidal silica added. The test program employed two groups of 100 mm cube specimens, where each group consisted of 12 concrete cubes.

Two embedded lengths (i.e., 10 and 20 mm) of *Type-A* fibres were included in the testing regime. Pull-out tests were conducted for fibres embedded in the reference concrete mix without colloidal silica (Mix 1) and the concrete mix containing colloidal silica (Mix 2). For Mix 1, the maximum pull-out force and average pull-out force of *Type-A* fibres with 10 mm embedment length were respectively 185 and 70 N, and the corresponding values of *Type-A* fibres with 20 mm embedment length were respectively 260 and 125 N. For Mix 2, the maximum pull-out force and average pull-out force of *Type-A* fibres with 10 mm embedment length were respectively 165 and 60 N, and the corresponding values of *Type-A* fibres with 20 mm embedment length were respectively 290 and 130 N. Therefore, the test results indicated no significant difference between Mix 1 and Mix 2 concrete specimens. The observed differences did not exceed 5%, which was insignificant considering the scatter of the results. These outcomes demonstrate that the application of *TCAM10* colloidal silica does not affect the bond behaviour of synthetic macro-fibres in concrete.

### 4.2. Use of Steel Micro-Fibres

**Figure 7:**
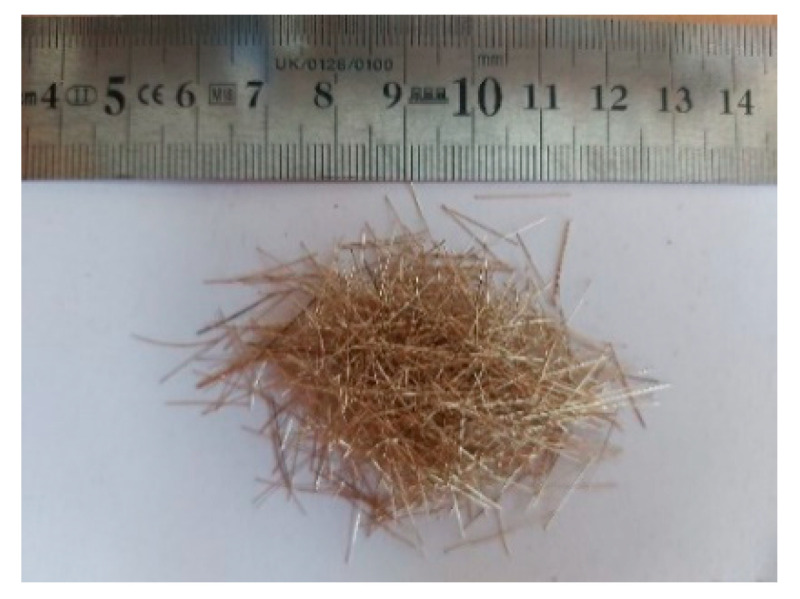
Dramix OL 13/.16 steel micro-fibres.

During the second attempt, the application of steel micro-fibres Dramix OL 13/.16 manufactured by Bekaert (Figure 7) was investigated for potential improvement of the bond performance of the synthetic macro-fibre [29,46]. The steel micro-fibres had a diameter of 0.16 mm and a length of 13 mm. The surface of the fibre was of a smooth texture and had a layer of protective copper coating applied in the factory. The fibre had a permissible tensile strength of 2000 MPa. The raw material of the steel micro-fibres was recycled from steel wires for the tire manufacturing industry. In this study, two volumetric contents of the steel micro-fibres (i.e., 0.5% and 1.0%) were dosed.

**Figure 8:**
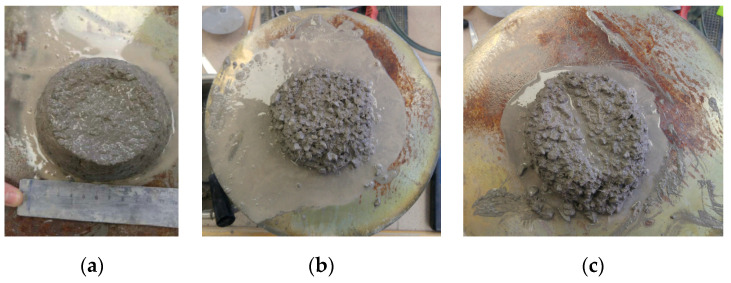
Slump tests during trial mixing: (**a**) 0.5% steel micro-fibre content with 2.2 kg/m^3^ superplasticiser; (**b**) segregation of paste in the concrete with 0.5% steel micro-fibres at an interim stage of trial mixing; (**c**) Mix 3b (Table 3).

Due to the rigidity of the fibre material, the presence of steel micro-fibres reduces the workability of concrete. Pilot trial suggested that the concrete mix in the first investigation stage could not be directly employed [37]. To attain adequate workability for proper casting and compaction, a concrete mix of better workability has to be used with the steel micro-fibres. In designing a suitable concrete mix for addition of steel micro-fibres, the reference concrete Mix 1 in Table 2 was adopted as the starting point for the trial. The slump of concrete measured in accordance with European Standard EN 12350-2:2011 in Section 2 was approximately 180 mm. Figure 8 depicts the trial mixing results. The initial addition of the steel micro-fibres reduced the slump to 110 mm (Figure 8a). The superplasticiser dosage was increased with an intent to restore sufficient workability. To avoid the segregation problem of the paste due to an excessive dosage of the superplasticiser (Figure 8b), other measures were adopted, which included reducing the maximum size of aggregates and angularity of aggregate particles. Accordingly, the mix proportioning was adjusted to achieve a consistent concrete mix (Figure 8c). The optimal superplasticiser dosage was obtained through a trial procedure, such that the concrete mixes had ample workability to ensure thorough mixing and uniform distribution of steel micro-fibres and, at the same time, there was no segregation problem.

Table 3 describes the final proportions and physical properties of the concretes adopted for steel micro-fibres addition. Among these, Mix 3a contained a fibre content of 0.5% by volume and Mix 3b contained a fibre content of 1.0% by volume. During concrete mixing, the solid ingredients except the steel micro-fibres were first mixed with half of the water and superplasticiser mixture, and then the fibres and remaining mixture of water and the superplasticiser were added and thoroughly mixed. Two concrete cube specimens were produced from each concrete mix for pull-out testing. Three synthetic macro-fibres were inserted in each cube, as described in Section 2, and only the results from the “side” fibres were considered. The pull-out tests were carried out at the concrete age of 30 days. Average compressive strength of the 100 mm concrete cubes cast from Mix 3, Mix 3a, and Mix 3b was equal to 54.7, 57.7, and 48.0 MPa, respectively.

**Figure 9:**
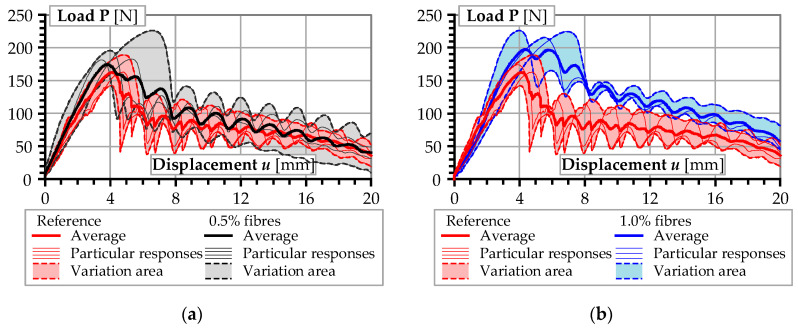
Comparison of the pull-out test results of reference concrete (red lines) and concrete containing steel micro-fibres (black lines): (**a**) with fibre content of 0.5%; (**b**) with fibre content of 1.0%.

Figure 9 shows the results of pull-out tests of the synthetic fibres embedded in concrete containing steel micro-fibres. Table 4 summarises the test results, including the maximum pull-out force **P***_max_* and the corresponding slip displacement *u_P_*, the total deformation energy *δ* (evaluated as the area beneath the load–displacement curve up to the maximum slip displacement), and the corresponding slip displacement *u_δ_*. The outcome from the broken fibre is highlighted in grey colour in the table. This result was excluded from Figure 9.

Table 4 illustrates the effect of the addition of the steel micro-fibres. There is a significant positive relationship between the fibre content and the deformation energy. On average, the deformation energy associated with the pull-out of the synthetic macro-fibre from the plain concrete was 1638.4 Nmm. The value of deformation energy increased to 1879.3 and 2389.4 Nmm, respectively, for 0.5% and 1.0% contents of the steel fibres. The maximum pull-out force **P***_max_* required to pull out the synthetic fibre also increased upon addition of steel micro-fibres. An increase in maximum pull-out force by 10% (from 175 to 190 N) and by 30% (from 170 to 225 N) corresponding to the fibre contents of 0.5% and 1.0% was obtained upon averaging the results across similar specimens. The bond stiffness of the synthetic macro-fibre was enhanced by the addition of steel micro-fibres. Taking the secant bond stiffness at peak pull-out force (equal to **P***_max_*/*u_P_*) as the basis of comparison, on average, the secant bond stiffness at peak pull-out force was increased by 25.9% and 26.8% upon adding 0.5% and 1.0% of steel micro-fibres, respectively. The increase in bond stiffness can effectively reduce the micro-crack width under pull-out action, which is advantageous to the structural performance of FRC.

## 5. Discussion of the Results

Reliable experimental results have been obtained from the series of pull-out testing. At this juncture, it is noteworthy that from the structural design perspective, the structural behaviour of FRC is dependent on the interaction between concrete and multiple distributed fibres as a composite, rather than the bond of a single fibre. Indeed, the number of fibres crossing a crack is a crucial factor determining the efficiency of the dispersed reinforcement. In this context, each 100 g batch of fibres approximately contains 3840 *Type-A* fibres or 6900 *Type-B* fibres. Though *Type-A* fibres were able to resist higher pull-out force per single fibre, the larger number of *Type-B* fibres were beneficial in promoting structural efficiency. The choice of fibre type for optimal performance in different structural applications will be a valuable topic for further research. Moreover, the structural efficiency of other types of synthetic macro-fibres could be examined by undergoing the test campaign considering the present research work as the reference.

The use of colloidal silica and steel micro-fibres in producing concrete specimens for the pull-out test yielded results with disparity, and an account of the interpretation is presented in the following. These reported findings are valuable since such experimental means had been rarely explored in the past. It was expected that the pore densification of colloidal silica could improve the matrix microstructure at the interface between the synthetic macro-fibre and concrete. However, the absence of pull-out force enhancement illustrated that the adhesion component of the bond [47] might not play a crucial role in resisting against the slip. During the experimentation, the dosage of colloidal silica recommended by the manufacturer and supplier was adopted. Nevertheless, increasing the dosage of colloidal silica may yield a positive effect. Further research is recommended for experimentation with a higher dosage.

On the contrary, the steel micro-fibres were capable of improving the bond strength of synthetic macro-fibres. The reason would be that the steel micro-fibres provided confinement against microstructural deformation in the concrete. The comparative analysis of Figure 6b and Figure 9b reveals essential similarity in the bond behaviour of synthetic macro-fibres in concrete. Proper proportions of steel micro-fibres and fine aggregates produced a comparable effect on the bond resistance as the application of coarse aggregates (the respective average deformation energy was equal to 2389.4 and 2671.7 Nmm). The reduction in the steel fibre content from 1.0% to 0.5% reduced the deformation energy released during the debonding of the synthetic macro-fibres from 2389.4 to 1879.3 Nmm, though the corresponding compressive strength of the concrete increased from 48.0 to 57.7 MPa (Table 3).

The above observations suggest that the mechanical interlock component [47] plays a predominant role in the pull-out resistance of synthetic fibres. There existed a distinct difference between the pull-out behaviour of metallic fibres and anchors [48,49,50], the underlying reason being the difference in material stiffness. The higher stiffness of the latter could mobilise the shear cone resistance of concrete and possibly lead to cone failure of concrete eventually. On the other hand, due to the low stiffness of synthetic macro-fibres, the slippage mechanisms are activated at a more advanced deformation stage, compared to steel fibres [6]. The reduction in the cross-section of the synthetic fibre due to the Poisson effect would diminish the bond interaction as well as the significance of the adhesion component.

Steel micro-fibres, through improving the mechanical properties of concrete [30] and mobilising a larger volume of concrete to anchor the macro-fibre against pull-out [51], could possibly exert synergetic effect with the synthetic macro-fibre. Such synergetic effect is worthwhile for more in-depth research. Overall, the present study has yielded useful results for developing efficient FRC.

There are some issues in concrete technology that deserve attention: the steel micro-fibres demand high workability of the concrete for proper mixing and uniform fibre distribution, and this affects the concrete mix proportioning. At a given water to cementitious materials ratio to satisfy the strength requirement, since an excessive dosage of superplasticiser would lead to the risk of segregation, the superplasticiser could not be added inadvertently. In this study, aggregates with reduced maximum aggregate size and less angularity were used. This was helpful in reducing the superplasticiser demand and successfully resulted in good quality steel micro-fibre concretes.

## 6. Conclusions

This manuscript has presented the investigation of the bond behaviour of synthetic macro-fibres. The experiment encompassed two types of synthetic macro-fibres available in the market. A bespoke gripping system newly developed to protect the fibres from local damage was employed for conducting the pull-out testing. The experimental campaign consisted of two stages. The mechanical performance of the bond was the research object in the first investigation stage. In the second investigation stage, one fibre type was chosen for examining the possibility of improving the bond performance. The following conclusions are drawn from the experimental findings:

- The loading capacity (the displacement energy *δ*) of the *Type-A* fibre was almost twice of that of the *Type-B* fibre. The average deformation energy *δ* dissipated for the length *l_e_* = 10 mm for *Type-A* and *Type-B* fibres was equal to 1077.4 and 533.9 Nmm; whereas for the length *l_e_* = 20 mm, the deformation energy for the two types of fibres was equal to 2671.7 and 1484.1 Nmm, respectively. Thus, *Type-A* fibres were chosen for further investigation of the feasibility to improve the bond performance.
- The application of colloidal silica with the dosage adopted in this study was found to be inefficient in improving the bond strength of synthetic macro-fibres in concrete. The observed differences in the maximum pull-out forces did not exceed 5%, which was insignificant (in the statistical sense) in consideration of the inherent scatter of results.
- The use of steel micro-fibres was an effective measure to improve the bond performance. By adding 0.5% and 1.0% of steel micro-fibres by volume of concrete, the maximum pull-out force of synthetic fibres increased by 10% and 30%, respectively, upon averaging the results across similar specimens. The respective values of deformation energy also increased by 15% and 45%. The steel micro-fibres improved the bond by possibly enhancing the mechanical interlock component of the slip resistance of the synthetic macro-fibre. Nevertheless, the use of steel micro-fibres requires special attention in the mix proportioning of concrete.
- The test results demonstrated that the mechanical interlock component of bond plays a predominant role in the pull-out resistance of synthetic macro-fibres. Proper proportions of steel micro-fibres and fine aggregates produced a comparable effect on the bond performance as the application of coarse aggregates (the respective average deformation energy was equal to 2389.4 and 2671.7 Nmm). The reduction in the steel fibre content from 1.0% to 0.5% reduced the deformation energy from 2389.4 to 1879.3 Nmm, though the corresponding compressive strength of the concrete increased from 48.0 to 57.7 MPa.

## Figures and Tables

**Table 1:** Summary of the pull-out test results ranged by deformation energy *δ*.

Fibre Type	*l_e_* [mm]	P*_max_* [N]	*u_P_* [mm]	*δ* [Nmm]	*u_δ_* [mm]
*A*	10	198	4.48	1175.3	10
*A*	10	213	5.02	1153.7	10
*A*	10	190	2.61	1080.2	10
*A*	10	198	4.86	1039.5	10
*A*	10	176	4.90	938.3	10
*A*	10	224	4.76	330.8	5.55
*A*	20	234	5.90	2933.1	20
*A*	20	220	6.77	2661.9	20
*A*	20	229	6.56	2651.2	20
*A*	20	210	7.87	2440.2	20
*A*	20	238	8.62	903.6	8.64
*A*	20	240	5.88	886.2	6.30
*A*	20	197	5.79	656.6	7.21
*B*	10	139	3.88	782.1	10
*B*	10	120	3.90	652.9	10
*B*	10	109	5.39	578.8	10
*B*	10	98	3.00	535.3	10
*B*	10	91	4.88	455.7	10
*B*	10	71	3.06	442.1	10
*B*	10	84	3.62	415.0	10
*B*	10	86	2.48	409.1	10
*B*	20	178	4.39	2003.6	20
*B*	20	150	3.40	1437.8	20
*B*	20	152	3.28	1010.8	20
*B*	20	119	4.01	372.8	5.25
*B*	20	141	4.74	267.3	4.95

**Table 2:** Mix proportions of concrete with colloidal silica [kg/m^3^].

Component	Mix 1 ^1^	Mix 2
Cement CEM I 42.5 R	298.7	298.7
Water	164.3	162.3
Gravel 4/16 mm	983.8	983.8
Sand 0/4 mm	783.7	783.7
Limestone	99.6	99.6
Colloidal silica *TCAM10*	–	2.0
Superplasticiser *Mapei Dynamon XTend*	2.234	2.234

^1^ The proportions are similar to the concrete used in Section 2.

**Table 3:** Mix proportions and physical properties of concrete with steel micro-fibres.

Component	Mix 3	Mix 3a	Mix 3b
Cement CEM I 42.5 R [kg/m^3^]	396.0	396.0	396.0
Water [kg/m^3^]	204.0	203.0	203.0
Gravel 2/5 mm [kg/m^3^]	960.0	945.0	945.0
Sand 0/4 mm [kg/m^3^]	1038.0	1022.0	1022.0
Limestone [kg/m^3^]	198.0	198.0	198.0
Superplasticiser *Mapei Dynamon XTend* [kg/m^3^]	6.0	7.0	7.0
Steel micro-fibres [kg/m^3^]	–	47.0	94.0
Slump [mm ^1^]	160	165	145
Compressive strength [MPa]	54.71	57.68	47.95

^1^ Slump of fresh concrete after dosing with steel micro-fibres.

**Table 4:** Summary of the bond improvement results of *Type-A* fibres with *l_e_* = 20 mm.

Fibre Content	P*_max_* [N]	*u_P_* [mm]	*δ* [Nmm]	*u_δ_* [mm]
–	142.8	4.01	1267.5	20
–	175	3.95	1523.6	20
–	163.8	4.79	1706.2	20
–	189.3	4.81	2056.4	20
0.5%	195.5	4.03	1481.7	20
0.5%	226	6.61	2394.2	20
0.5%	181.7	3.53	1713.5	20
0.5%	183	5.35	1927.9	20
1.0%	225.2	6.96	2289.7	20
1.0%	183.2	2.92	363.2	3.18
1.0%	226.7	4.03	2589.7	20
1.0%	215.8	5.59	2288.7	20
